# Case report: Treatment of psychiatric symptoms for an acromegalic patient with pituitary adenoma

**DOI:** 10.3389/fpsyt.2022.1068836

**Published:** 2022-12-01

**Authors:** Zhongyong Shi, Enzhao Cong, Yan Wu, Xinchun Mei, Yun Wang, Daihui Peng

**Affiliations:** ^1^Division of Mood Disorder, Shanghai Mental Health Center, Shanghai Jiao Tong University School of Medicine, Shanghai, China; ^2^Anesthesia and Brain Research Institute, Tongji University School of Medicine, Shanghai, China

**Keywords:** acromegaly, pituitary tumor, mental disorders, Aripiprazole, case report

## Abstract

Acromegalic patients always demonstrate a wide range of clinic manifestations, including typical physical changes such as acral and facial features, as well as untypical neuropsychiatric and psychological disturbances. However, there is still a lack of clinical guidance on the treatment for acromegalic patients with psychiatric comorbidities. We therefore share this case to provide a reference for clinicians to manage the acromegalic patients with psychiatric symptoms. This case report describes a 41-year-old male with an 8-year history of acromegaly due to growth hormone-secreting pituitary adenoma, the maximum cross-sectional area of which was 42 mm × 37 mm demonstrated by pituitary magnetic resonance imaging (MRI). The patient received conservative medicine treatment by regularly injecting with Sandostatin LAR 10 mg per month. Two days before admission, he suddenly presented with an acute psychotic episode. In addition to the typical acromegaly-associated changes, his main clinical presentations were olfactory/auditory hallucinations, reference/persecutory delusions, instable emotion and impulsive behavior. Considering the schizophrenic-like psychoses and course features, he was diagnosed with Brief Psychotic Disorder according to Diagnostic and Statistical Manual of Mental Disorders, 5th edition (DSM-5) after a multidisciplinary consultation and evaluation. He was prescribed Aripiprazole, which had less extrapyramidal symptoms and minimal influence on prolactin elevation, with the dose of 5 mg per day to control the psychiatric symptoms and he responded quite well. At the time of discharge and the follow-up 2 month later, the patient was stable without recurrence of any psychotic symptoms. The levels of growth hormone (GH) and insulin-like growth factor 1 (IGF-1) 1 week after discharge were 2.22 ng/mL [normal range (0–2.47 ng/mL)] and 381 μg/L [normal range (94–284 μg/L)], respectively, which were similar to those before the psychotic episode. Results from this report further supported that small dose of Aripiprazole had little influence on hormonal levels and the development of pituitary macroadenoma. This particular case emphasizes the importance for the clinician to master and carefully identify the possible symptoms of mental disorders associated with acromegaly, and also highlights the need for further investigation in more efficient treatment strategies for acromegalic cases with psychiatric comorbidities.

## Introduction

Acromegaly is a rare and chronic illness, typically characterized by changes in appearance, skeletal deformities, and metabolic disorders, caused by abnormally high levels of growth hormone (GH) and insulin-like growth factor 1 (IGF-1). The clinical symptoms of acromegalic patient are mostly (95%) attributed to chronic hypersecretion of GH and pituitary adenoma (1). Diagnosis of acromegaly includes measurements of IGF-1, followed by oral glucose tolerance testing (OGTT) for GH suppression and magnetic resonance imaging (MRI). Specifically, IGF-1, secreted by liver due to the binding of growth hormones and their receptors, is recommended for patients with typical clinical symptoms of acromegaly (2). The estimated annual incidence rates range between 2 and 11 cases per million, which is a statistically abnormal phenomenon (3). It has been reported that the life expectancy of acromegalic patients is reduced by 30%, and they have 2.4- to 4.8-fold increased mortality rate as compared to the general population, mostly attributed to the complications of cardiovascular, cerebrovascular, and pulmonary dysfunction (4).

Acromegalic patients demonstrate a wide range of clinical manifestations, in addition to the typical changes (acral and facial features), some of which can be neuropsychiatric and psychological disturbances. Limited studies have been carried out to describe emotional and behavioral alterations with acromegaly, suggesting that acromegalic patients suffer from an increased prevalence of mental disorders and a higher risk of maladaptive personality traits (5, 6). In contrast, other studies demonstrated that psychiatric symptoms were likely to be presented before the acromegaly diagnosis in most acromegalic cases, and the average time span between the onset of mental disorders and the acromegaly diagnosis ranged from 9.4 to 22.4 years (6). It is unknown whether the diagnosis of mental disorders was an indicator or a preclinical stage of the disease status for acromegaly. Nevertheless, the coexistence of both psychopathological and acromegalic features might indicate the presence of a causal relationship between mental disorders and acromegaly, which remains to be determined. Therefore, it is of great clinical significance to advocate early diagnosis and effective treatment of acromegaly with mental disorders as soon as possible in order to prevent further damage and improve long-term prognosis.

So far, there is still a lack of clinical guidance on how to optimize the treatment for acromegalic patients with neuropsychiatric comorbidities. In this report, we present a case of acromegaly accompanied with schizophrenic-like psychoses, and the purpose is to propose suggestions and precautions for the psychopharmacological treatment.

## Case history

### Case presentation

**FIGURE 1:**
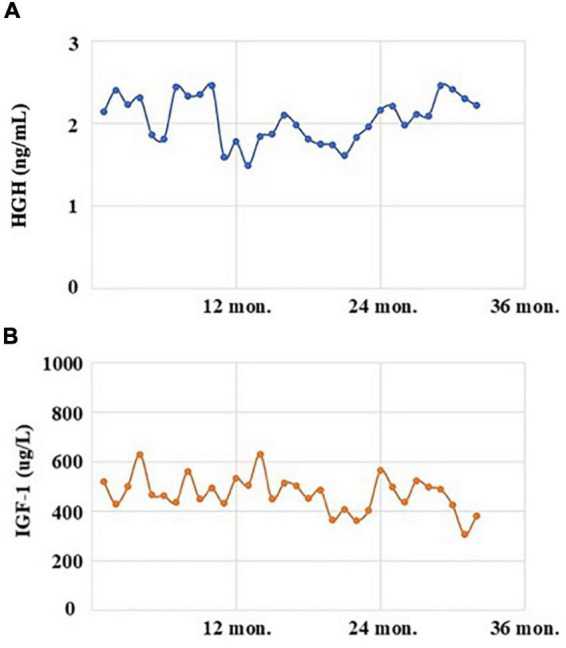
The levels of human growth hormone (HGH, normal range: 0–2.47 ng/mL; **A**) and insulin-like growth factor 1 (IGF-1, normal range: 94–284 μg/L; **B**) of the acromegalic patient with the treatment of Sandostatin LAR in recent 3 years.

**FIGURE 2:**
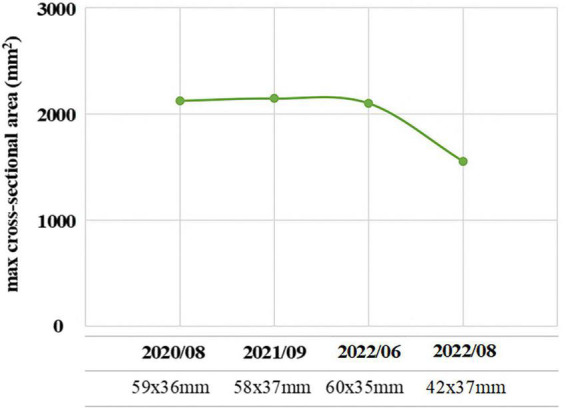
The maximum cross-sectional area (mm^2^) of the pituitary macroadenoma measured by pituitary magnetic resonance imaging (MRI) of the acromegalic patient with the treatment of Sandostatin LAR in recent 3 years.

The patient, a 41-year-old male, married, Han nationality of Chinese, had a history of acromegaly for 8 years. At the beginning, he came to see a doctor with the complaints of eyesight loss in his right eye. Then, he was recommended to Department of Neurosurgery in a famous general hospital in Shanghai, China. After conducted completed examinations, he finally got a definite diagnosis of acromegaly attributed to GH-secreting pituitary macroadenoma. At the time of diagnosis, the invasive pituitary adenoma had compressed the optical chiasm and invaded the surrounding tissues demonstrated by the patient’s pituitary MRI. After required brain surgery consultation, a surgical approach was not considered necessary mainly because of high risk and cost, and then medical intervention with Sandostatin LAR (Octreotide acetate microspheres for injection) was started. The patient received the injection of Sandostatin LAR 10 mg per month with regularly monitoring of GH/IGF-1 levels (Figure 1) and pituitary MRI (Figure 2) at Department of Endocrinology of the same hospital. In general, his physical condition remained stable except for the lost eyesight, which was irreversible due to the delayed diagnosis and intervention.

On August 3, 2022, he suddenly developed acute psychotic symptoms of hallucination, paranoid ideation, seriously impulsive and aggressive behaviors. His family initially took him to Department of Emergency in the original general hospital. According to his preliminary laboratory and imageological examinations, there was no sufficient evidence indicating the possibility of patient’s psychotic symptoms due to severe sepsis, intracerebral hemorrhage or other acute organic diseases. Since the patient’s GH/IGF-1 levels have been stable in recent 3 years, and brain imaging didn’t suggest exacerbation of original pituitary macroadenoma, whether there was a causal relationship between the psychosis episode and the pituitary remained to be further investigated in a longer time. Therefore, after a multidisciplinary consultation and evaluation, the patient was later referred to our hospital, a specialized psychiatric hospital in Shanghai, China, on August 5, 2022.

Upon admission, the patient additionally reported a history of hypertension for 3 years, which had been controlled stable by taking Nifedipine controlled-release tablet 30 mg per day. He reported no history of heart or respiratory disease, diabetes, epilepsy, or drug allergy. There was no other personal or familial history of physical illness or psychosis.

### Investigation

**FIGURE 3:**
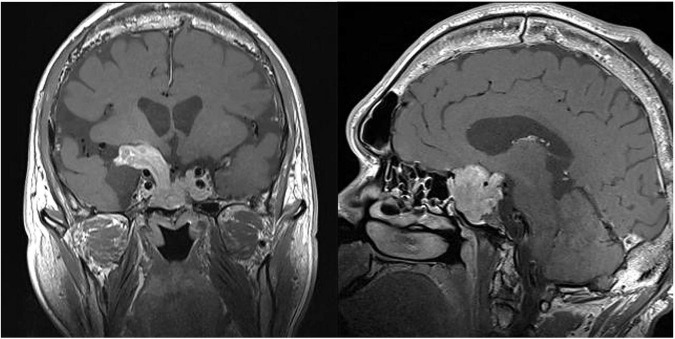
The magnetic resonance imaging (MRI) of coronal **(left)** and sagittal **(right)** sections showing a pituitary macroadenoma. Specifically, the maximum cross-sectional area was 42 mm × 37 mm, which had compressed the optical chiasm, invaded the skull base bone, and surrounded blood vessels in sellar area.

Physical examination showed typical characteristic changes in the patient’s appearance, such as enlarged hands and feet, prognathism (excessive protrusion of the mandible or maxilla), enlargement of the supraorbital ridges, the tongue, the nose, and thickening of the lips. Moreover, visual field perimetry demonstrated a hemianopsia in his right eye because of optic chiasm compression by the enlarged pituitary adenoma. The patient’s contrast-enhanced MRI of the pituitary gland revealed a pituitary macroadenoma and the maximum cross-sectional area was 42 mm × 37 mm, which had compressed the optical chiasm, invaded the skull base bone, and surrounded blood vessels in sellar area. Furthermore, his frontal bone, parietal bone and occipital bone were detected diffusely thickening and ballooning (Figure 3). The level of GH was 2.30 ng/mL [normal range (0–2.47 ng/mL)], and the level of IGF-1 was 307 μg/L [normal range (94–284 μg/L)], which were examined on July 20, 2022, before the psychosis episode. In addition, the levels of serum myocardial enzymes were elevated: lactate dehydrogenase (LDH) 2,52I U/L [normal range (120–250 IU/L)], creatine kinase (CK) 1,788 U/L [normal range (50–310 U/L)], creatine phosphokinase MB (CK-MB) 46 U/L [normal range (0–25 U/L)]. The electrocardiogram (ECG) indicated sinus tachycardia with a heart rate of 122 beats per minute. Since the patient didn’t have history of heart disease, didn’t complain of any serious symptoms, such as chest pain or pressure, severe shortness of breath or dizziness, or faintness, and there was no indication of acute myocardial infarction of his ECG, cardiovascular diseases could be eliminated. The most probable reason for the elevation of enzymes was attributed to skeleton muscle injury because the patient was extremely agitated and overactive before admission. Results of other laboratory tests including blood/urine/stool routine, liver and renal function, electrolytes, thyroid function, prolactin level and auxiliary examinations were without obvious abnormalities. Further psychiatric examination found that the patient was conscious with normal orientation. Olfactory and auditory hallucinations were observed. It was noted that he had apparent reference/persecutory delusions, sense of being tracked and monitored. Moreover, his emotion was irritable, and behavior was disturbed, so that he assaulted his parents since he thought they would harm and deceive himself.

### Diagnosis

First of all, for an adult male presented with first-episode acute psychiatric symptoms, it is prior to include organic diseases in differential diagnosis of functional mental disorders. But there was no disturbance of consciousness in this patient. Moreover, he had completed necessary examinations and received multidisciplinary consultation and evaluation in a general hospital before being admitted to our hospital, evidence for the psychotic symptoms due to the progression of pituitary macroadenoma or other organic diseases was insufficient at the moment. Secondly, the patient had no previous history of smoking, alcohol drinking, drug or caffeine abuse, and the results of his urine drug screening in hospital were negative. In addition, he had regularly injected with Octreotide (somatostatin receptor ligands, SRLs; the first-line drug for medical intervention in persistent acromegalic patients) for several years, but no side effects were reported. Hence, his psychiatric symptoms due to psychoactive substances or non-addictive substances could also be excluded. Collectively, considering the patient’s schizophrenic-like psychoses and course features, he was diagnosed with Brief Psychotic Disorder according to the criteria of DSM-5. However, it is necessary to follow the patient’s clinical manifestations, GH/IGF-1 levels and prognosis for a longer time in the future to confirm the diagnosis and whether there is a causal relationship between the psychiatric symptoms and the probably progressing pituitary adenoma in this acromegalic patient.

### Therapeutic intervention

Aripiprazole was prescribed to the patient with an initial dose of 5 mg per day. About 3 days later, the patient’s hallucinations and delusions partially decreased, and his agitated behaviors also significantly improved. Reexamination of ECG and serum myocardial enzymes demonstrated normal results. After 1 week, the patient’s psychiatric symptoms totally disappeared, and he was discharged from our hospital and would continue with his regular treatment for acromegaly.

### Outcome and follow-up

Two months later, this patient accepted our follow-up by a phone call. He was sticking with the medication treatment of Aripiprazole 5 mg per day, regularly injected with Octreotide as before, and he remained in stable condition without recurrence of any psychotic symptoms. The levels of GH and IGF-1 after discharge on August 19, 2022, were 2.22 ng/mL [normal range (0–2.47 ng/mL)] and 381 μg/L [normal range (94–284 μg/L)], respectively, which indicated that treatment with small dose of Aripiprazole had not significant influence on the hormonal levels.

## Discussion

Acromegaly is a relatively rare disease associated with excessive production of GH by the adenoma. The disease was initially described by Pierre Marie in 19th century, which has been the subject of interest for centuries (7). The clinical presentations of acromegalic patients are always insidiously, causing significant diagnostic delay by five additional years since the symptom onset (3). A retrospective study with 324 acromegalic patients revealed that 96.3% of the patients were not diagnosed until serious acromegaly-associated comorbidities such as facial and acral changes had developed, resulting in poor prognosis and higher healthcare burden (8). Consistently, for the acromegalic case in this report, the pituitary adenoma had developed to be macroadenomas at the time of detection, which caused the patient’s irreversible eyesight loss, increased great risk for adverse surgical outcomes, and even let the patient miss the opportunity for brain surgery. It has also been reported that some acromegalic patients would demonstrate with neuropsychiatric disturbances at the early stage of disease, and therapy with dopamine antagonists might precede the diagnosis of acromegaly. However, very little was found in previous studies on the treatment options for mental disorders in acromegalic patients with pituitary adenoma.

In clinical practice, it is necessary to master and carefully identify the possible symptoms of mental disorders associated with acromegaly, in order to improve the efficiency of diagnosis and reduce the rate of misdiagnosis. The current case report described the effective treatment for an acromegaly patient diagnosed with Brief Psychotic Disorder, which can provide a reference for clinicians to manage the acromegalic patients with psychiatric comorbidities.

### Acromegaly and mental disorders

A wide range of neuropsychiatric symptoms have been reported in acromegalic patients, including neurocognitive complications, psychiatric and psychological symptoms, alteration in personality and relevant somatic comorbidities (9). As mentioned in the literature review, the prevalence of neurocognitive dysfunctions was 2–33% in acromegaly, mostly affecting memory and attention (10). The prevalence of psychiatric disorders in acromegaly reached 63%, mostly involving depression, followed by psychosis and anxiety (11). Specifically, psychiatric morbidity according to General Health Questionnaire-28 (GHQ-28), mainly anxiety and insomnia, occurs in 50% of acromegalic patients (12). Sievers et al. conducted a cross-sectional study to investigate the prevalence of mental disorders among 81 patients with acromegaly (6). Results consistently showed that the lifetime prevalence of mental disorders in acromegalic group was 45.7%, and the 12-month prevalence was nearly three times higher than normal control subjects (6). Another observational study with larger sample size (*N* = 115) demonstrated that mental disorders were diagnosed in 79.1% of acromegalic patients and schizophrenia spectrum disorders were found in 4.3% which significantly exceed that in the general population (13).

However, evaluation of psychiatric or psychological symptoms has revealed inconsistent findings. In addition to the differences in the prevalence of psychiatric morbidities among acromegalic patients, some earlier studies were unable to demonstrate a relationship between the severity of psychopathological symptoms and alterations in GH levels (14). The contradictory findings might attribute to the methodological problems, lack of standardized evaluation techniques, heterogeneous study populations or insufficient sample sizes. It is worth highlighting that standardized evaluation and dynamic monitoring of acromegaly with an interdisciplinary approach, such as psychological and psychiatric consultation is needed.

There are many possible causes of psychological or psychiatric disorders combined with acromegaly, which might be related to the impaired body image, brain structure changes particularly the extension of adenoma to the fronto-temporal region, pituitary adenomas associated hormonal disturbance (hyperpituitarism and hypopituitarism), as well as the treatment with dopamine agonists. Specifically, it has been evidenced that patients with active untreated acromegaly suffer mental disorders, which are associated with irreversible effects of high GH/IGF-1 levels on central nervous system, and their severity increases according to hormone hypersecretion. Sievers et al. firstly demonstrated a disturbed macroscopic brain architecture, e.g., increased total gray and white matter volumes, in acromegaly by *in vivo* high-resolution MRI studies (15). Additionally, associations have also been reported between time delay of diagnostic process and psychosocial impairment (16). The acromegalic case in this report mainly presented with apparent hallucinations and delusions, as well as secondary behavioral disturbances. It remains unclear whether long-term GH and IGF-1 excessively secreting alters the psychopathological risk by influencing brain morphology or function in such acromegalic case.

### Acromegaly and antipsychotic treatment

Treatment aims for acromegaly mainly include normalization of GH and IGF-1 levels, destruction or excision of tumor size, and reduction of acromegaly-associated comorbidities (17). Current therapeutic interventions include transsphenoidal surgery (gold standard), radiotherapy and medical intervention (18). However, acromegalic patients with neuropsychiatric symptoms are still confronting with a stubborn therapeutic challenge at the present time.

Prior studies have shown that some acromegalic cases initially present with mental disorders long before being diagnosed and treat with antipsychotics, some of which might promote the disease progression. Koroglu et al. reported a case of paranoid schizophrenia who was prescribed risperidone for 14 years and was finally diagnosed of acromegaly with pituitary macroadenoma (19). Iglesias et al. also reported three cases of schizophrenic patients treated with antipsychotics (paliperidone, amisulpride, clozapine, and haloperidol) for several years who finally developed acromegaly due to a GH-secreting pituitary macroadenoma (20). They considered that schizophrenia and/or its antipsychotic therapy with dopamine antagonists in the long term might be in relation with the development of acromegaly, and the potential pathophysiological mechanisms were related to the alterations in dopaminergic neurotransmission due to psychiatric disease itself or its pharmacological treatment.

Dopamine is a neurotransmitter produced in several areas of the brain and plays multiple functions. Specifically, dopamine is a precursor of norepinephrine, which can increase and inhibit GH secretion via α- and β-adrenergic pathways, respectively. There were some earlier studies reporting that Chlorpromazine was effective in markedly decreasing the concentration of serum GH and improving clinical manifestations in acromegalic patients (21). The potential explanations of chlorpromazine acting in acromegaly were considered to be associated with its effects of adjusting the adrenergic activity in central nervous system and consequently reducing the level of GH, but further cohort studies were needed to clarify the mechanisms.

Currently, the relationships between antipsychotics and pituitary tumors have drawn public attention (22). In clinical practice, atypical antipsychotic drugs have become common choices particularly in the treatment of psychotic disorders for less extrapyramidal side effects and well tolerance. However, the endocrinological side effects, especially the associated hyperprolactinemia and pituitary tumors, have been frequently observed. An analysis of the European pharmacovigilance database (EudraVigilance) was carried out to assess the association between antipsychotics and pituitary, the highest proportional reporting ratio values were found for Amisulpride, then Risperidone and Paliperidone. Sulpiride and Haloperidol showed a higher risk among typical antipsychotics (23). Similarly, Szarfman et al. conducted a retrospective pharmacovigilance study to analyze the disproportionality of seven widely used antipsychotic medications (Aripiprazole, Clozapine, Olanzapine, Quetiapine, Risperidone, Ziprasidone, and Haloperidol) with pituitary tumors by screening the US Food and Drug Administration (FDA) Adverse Event Reporting System (AERS) Database (24). Results demonstrated that Risperidone had the strongest association with pituitary tumors, followed by Haloperidol, Ziprasidone, and Olanzapine, while the association for Clozapine and Quetiapine was relative weak. Specifically, the most important clinically relevant finding was that the use of Aripiprazole would not increase the risk of pituitary tumors. It has been explained that the affinities for occupying and blocking the D_2_ receptors correspond to the strength of the association between antipsychotics and pituitary tumors (25). It is important to note that Aripiprazole has a unique pharmacological profile for its weak partial agonist activity at the D_2_ receptors, with less extrapyramidal symptoms and minimal prolactin elevation. As for the current case of acromegaly, we thus chose Aripiprazole for the treatment of psychiatric symptoms in order to avoid aggregating the procession or reoccurrence of pituitary macroadenoma itself. Results from this case further supported that small dose of Aripiprazole had little influence on the fluctuation of GH and IGF-1 levels, which might be one of the appropriate choices for the treatment of acromegaly with mental disorders. These findings raise the need for clinical awareness and strengthen the importance for larger cohort studies with a longer follow-up to validate the optimized psychopharmacological treatments and to ensure the generalization of research findings.

### Limitations

On one hand, the follow-up time was relatively short to see the long-term influence for the current antipsychotic treatment on hormonal levels of the acromegalic patient. On the other hand, the findings from a single case may not apply to general patients, which limit the significance in clinical practice. Further randomized controlled trials (RCT) are pending to verify the effects or safety for the psychopharmacological treatments in acromegaly with neuropsychiatric comorbidities.

## Conclusion

Since only few reports on acromegalic patients accompanied with prominent mental disorders are available, experiences summed up from herein case are as follows: (1) Clinicians should pay attention to the diagnosis and treatment of mental disorders in acromegalic patients, GH/IGF-1 levels and pituitary MRI should be regularly evaluated when antipsychotics are prescribed. (2) Moreover, it is important for psychiatrist to keep an eye on the presence of morphological modifications in order to make early diagnosis for acromegalic patients. (3) Treatments for acromegaly always require a teamwork of neurosurgeons, endocrinologists, radiation oncologists and psychiatrists, it is valuable of a multidisciplinary evaluation and management. Conclusively, this enlightening case might inspire clinicians to further study the causal relationship between mental disorders and acromegaly, and to investigate more efficient treatment strategies for similar cases.

## Data availability statement

The original contributions presented in this study are included in the article/supplementary material, further inquiries can be directed to the corresponding authors.

## Ethics statement

Ethical review and approval was not required for the study on human participants in accordance with the local legislation and institutional requirements. The patients/participants provided their written informed consent to participate in this study.

## Author contributions

ZS prepared the study design and drafted the manuscript. EC, YWu, and XM critically revised and edited the manuscript. YWang and DP contributed to the study design and revision for important intellectual content. All authors contributed to the article and approved the final submitted version.
